# Mortality in New-York City

**Published:** 1847-09

**Authors:** 


*Mortality in New-York City.*—The Annalist, August 1st, states that the deaths in the city and county of New-York from July 10th, to 23d, were 960. Cholera Infantum, 131; Cholera Morbus, 51; Typhoid and Typhus fever, 92; Fever, 22; Puerperal fever, 14. At the Hospital on Staten Island, the number remaining on the 12th July, was 709, since when, 156 patients have been admitted, 242 discharged, and 24 died, leaving 599 remaining: laboring under bilious remittent fever, 3; remittent fever, 284; typhus 182; small pox, 2; and other diseases, 112. On July 16, there were 800 patients of the Bellevue Hospital. The Grand Jury, having made an official visitation of the latter institution, reported in terms of great satisfaction upon the appearance of cleanliness and neatness, and that serious mortality from ship fever has been arrested. Much credit is attributed to the new resident Physician, Dr. Reese, for the improved condition of the hospital and inmates.

